# LncRNA Neat1 mediates miR-124-induced activation of Wnt/β-catenin signaling in spinal cord neural progenitor cells

**DOI:** 10.1186/s13287-019-1487-3

**Published:** 2019-12-18

**Authors:** Yi Cui, Yanyun Yin, Zhifeng Xiao, Yannan Zhao, Bing Chen, Bin Yang, Bai Xu, Hongwei Song, Yunlong Zou, Xu Ma, Jianwu Dai

**Affiliations:** 1 Reproductive and Genetic Center of National Research Institute for Family Planning, Beijing, 100081 China; 2 Key Laboratory of Molecular Developmental Biology, Institute of Genetics and Developmental Biology, Chinese Academy of Sciences, 3 Nanyitiao, Zhongguancun, Beijing, 100080 China; 3 Orthopaedics Surgery Department, China-Japan Union Hospital of Jilin University, 126 Xiantai Street, Changchun, 130033 Jilin Province China; 4 EHBIO Gene Technology, No. 46, Jiugulou Street, Beijing, 100100 China

**Keywords:** miR-124, Neat1, Spinal cord neural stem cells (SC-NPCs), Spinal cord injury (SCI)

## Abstract

**Background:**

Emerging evidence suggests that miR-124 performs important biological functions in neural stem cells (NSCs); it regulates NSC behavior and promotes the differentiation of NSCs into neurons, but the exact molecular mechanism remains unknown. And also, the role of miR-124 during spinal cord injury regeneration is unclear.

**Materials and methods:**

In order to explore the function of miR-124 in neural differentiation, the molecular markers (Tuj1, Map2, and GFAP) correlated with the differentiation of NSCs were detected by immunofluorescence staining both in cultured mouse spinal cord progenitor cells (SC-NPCs) and in spinal cord injury (SCI) animal models. The migration ability and apoptosis of cultured SC-NPCs were also evaluated by Transwell migration assay and TUNEL assay. In addition, the relative expression of lnRNA Neat1- and Wnt/β-catenin signaling-related genes were detected by quantitative real-time PCR.

**Results:**

In this study, we revealed that lncRNA Neat1 is involved in regulating Wnt/β-catenin signaling that is activated by miR-124 in SC-NPCs. LncRNA Neat1 was also found to play an important role in regulating neuronal differentiation, apoptosis, and migration of SC-NPCs. Furthermore, we demonstrated that overexpression of miR-124 resulted in elevated Neat1 expression, accompanied with the functional recovery of locomotion in a mouse model of spinal cord injury.

**Conclusions:**

Our results confirm the therapeutic effectiveness of miR-124 on the functional recovery of injured spinal cord, supporting the rationale and feasibility of miR-124 for spinal cord injury treatment in future clinical therapy. Furthermore, we concluded that the miR-124-Neat1-Wnt/β-catenin signaling axis is involved in regulating the cell function of SC-NPCs, and this may offer novel therapeutic avenues for future treatment of SCI.

## Background

Spinal cord injury (SCI) is a devastating disease that results in paralysis, which seriously affects patients’ quality of life. Because the spinal cord does not possess the capacity to regenerate, the restoration of bodily functions after SCI is always a major clinical challenge. The discovery of endogenous spinal cord neural stem cells (NSCs) at injury sites opened a new avenue for SCI therapy [1, 2]. It is well known that NSCs possess the ability to regenerate; therefore, the differentiation and migration abilities of endogenous NSCs may be important in functional recovery following SCI. Given that most endogenous NSCs tend to differentiate into glia rather than neurons, many studies have focused on ways to promote spinal cord neural progenitor cells (SC-NPCs) to differentiate into functional neurons [3, 4].

Increasing evidence has suggested that miR-124 is critical for neurogenesis and is involved in determining the neuronal fate of NSCs. Previous studies demonstrated that NSCs transfected with miR-124 were beneficial in the treatment of SCI rats compared with control NSCs [5, 6]. In agreement with these previous studies, the Basso Mouse Scale (BMS) scores in our study were higher in the miR-124 group compared with the control group at 1 or 2 weeks post-injury, demonstrating that overexpression of miR-124 at the injury site by exogenous injection is beneficial for functional recovery in an animal model of SCI. Compared with the control group, the immunofluorescence staining detection indicated that the expressions of Tuj1 and Map2 at the injury site were increased in the miR-124 group, whereas the expression of astroglial marker GFAP was decreased in the miR-124 group. The effect of miR-124 on promoting SC-NPCs to differentiate into neurons is thought to be at least partly related to its positive effect in SCI mouse models.

Elucidating the exact mechanism by which miR-124 affects NSCs may help to develop new therapeutic strategies for treating nervous system diseases. Many long non-coding RNAs (lncRNAs) have been shown to exhibit dynamic expression patterns during NSC differentiation and neurogenesis, and emerging evidence has demonstrated that lncRNAs play pivotal roles in regulating self-renewal and differentiation of NSCs [7–9]. Recently, many studies have suggested that lncRNAs are implicated in complex molecular circuitry in the neural differentiation processes. However, the underlying mechanisms in regulating NSC differentiation have not been well elucidated. In this study, we aimed to investigate the potential roles of lncRNA nuclear-enriched abundant transcript 1 (Neat1) in the regulation of NSC behavior that is influenced by miR-124.

As an epigenetic regulator, the research on lncRNA Neat1 attracted much attention, which exerts a very important role in the formation of paraspeckle. A growing body of evidence indicates that Neat1 play important roles in modulating multiple biological processes in immune cells, carcinoma cells, macrophages, and so on [10–13].. Nevertheless, little is known about how Neat1 regulates NSC behavior and function. Here, we discovered that miR-124 affected the expression of this lncRNA. The expression of Neat1 increased when miR-124 was overexpressed; in contrast, Neat1 expression was downregulated when miR-124 was knocked down by inhibitor. Furthermore, we demonstrated that Neat1 was implicated in the regulation of Wnt/β-catenin signaling activated by miR-124. Several studies have proved that Wnt/β-catenin signaling plays a key role in regulating the differentiation and migration of NSCs.

In summary, this study revealed that the expression of the lncRNA Neat1 could be affected by miR-124 and that miR-124 overexpression promoted NSC migration and induced neuron-specific differentiation. In addition, Neat1 may also promote the differentiation and migration abilities of NSCs, at least partly by activating Wnt/β-catenin signaling. Collectively, our work indicates a promising therapeutic potential for the miR-124-Neat1-Wnt/β-catenin signaling axis in the restoration of spinal cord function in SCI patients.

## Methods

### Culture and generation of neurospheres

The spinal cords were dissected from newborn C57BL/6 mice embryos (< 12 h after birth) to generate NPCs as previously published [14]. The spinal cords were cut up by scissors and then digested with TrypLE Express at 37 °C for 25 min. The single-cell suspensions were then resuspended in growth medium containing DMEM/F12 basal medium supplemented with 1% (v/v) penicillin-streptomycin solution, 2% (v/v) B-27 Supplement, 20 ng/mL epidermal growth factor (EGF), and 20 ng/mL recombinant human fibroblast growth factor (bFGF). For the follow-up experiments, the neurospheres were trypsinized (as previously described) [15] and seeded into polylysine-coated well plates or slides with high-glucose DMEM medium containing 10% fetal calf serum for adherent.

### Cell transfection

Small interfering RNAs (siRNAs) targeting NEAT1 and the negative control were obtained from Riobio Co., Ltd. (Guangzhou, China). The NEAT1 overexpression plasmid vector was constructed by Genearray Biotechnology (Shanghai, China). Transient transfection of siNeat1(100 nM) and plasmid vector (10 nM) was performed using Lipofectamine 3000 (Thermo Fisher, CA, USA) according to the manufacturer’s instructions. The empty vector and negative control of siRNA were also used as control. Transfected cells were harvested at 48 h after transfection for subsequent analysis and detection.

### Immunofluorescence staining

Immunofluorescence staining analysis was performed as described previously [15–17] (Additional file 1). The expression of Tuj1, Map2, and GFAP were detected in frozen sections of SCI tissue and SC-NPCs. The frozen sections and fixed cells were incubated with anti-Tuj1 rabbit polyclonal antibody (ab18207, 1:500, Abcam, Cambridge, UK), anti-MAP2 rabbit polyclonal antibody (ab32454, 1:500, Abcam, Cambridge, UK), or anti-GFAP (ab7260, 1:500, Abcam, Cambridge, UK) overnight. Subsequently, the samples were incubated with secondary antibodies: Alexa Fluor™ donkey anti-rabbit IgG (A21206, 1:500, Thermo Fisher, CA, USA). The cell nuclei were counter-stained with Hoechst 33342 (1:500; 94403, Thermo Fisher, CA, USA). The fluorescent images were observed with a Leica TCS SP8 scanning laser confocal fluorescence microscope (Leica Microsystems, Inc., Germany). The positive cells of Tuj-1, Map2, and GFAP and the measurement of neural length were quantified by using Image-Pro Plus software (Media Cybernetics).

### Quantitative real-time PCR

Total RNA was isolated from the tissue or cells using TRIzol (Thermo Fisher, CA, USA) according to the manufacturer’s instructions. For miRNA detection, total RNA was reverse-transcribed using the TaqMan miRNA Reverse Transcription Kit and specific miR-124 primers (Thermo Fisher, CA, USA). For mRNA detection, total RNA was reverse-transcribed using the TransScript One-Step gDNA Removal and cDNA Synthesis SuperMix (TransGen Biotech, Beijing, China). Next, quantitative RT-PCR was performed using mmu-miR-124-specific probes and specific gene primers (listed in Table 1) on a CFX96™ Real-Time PCR Detection System (Bio-Rad). Data were normalized using U6 and GAPDH expression levels, and the fold change compared with the control group values was calculated using the 2^−ΔΔCT^ method.

**Table 1:** The primer sequence of gene

Gene symbol	Primer sequence
RXRa-F	5′-CGAGCCATTGTCCTGTTC-3′
RXRa-R	5′-TGTGTTTGCAGTACGCTT-3′
Wisp1-F	5′-ATCCTGGGTATTTCTGCCT-3′
Wisp1-R	5′-ACAACTATGGTCCTCACCT-3′
Wnt5a-F	5′-AAGCATTTATATACAGGCGGT-3′
Wnt5a-R	5′-GAGCACAAAGAAACAGGACT-3′
DKK1-F	5′-GCTTGCAGGATACAGAAAGAT-3′
DKK1-R	5′-TAGACTGTCGGTTTAGTGTCTC-3′
Wnt2-F	5′-TCAGCAGAGGTCATATTCGC-3′
Wnt2-R	5′-TCCAGTGTCCTTGAAGATGTAA-3′
Neat1-F	5′- GGCAGGTCTAGTTTGGGCAT-3′
Neat1-R	5′-CCTCATCCCTCCCAGTACCA-3′

### TUNEL analysis

The SC-NPCs from all groups were washed with PBS and then fixed using 4% paraformaldehyde. The terminal transferase-mediated dUTP fluorescein nick-end labeling (TUNEL) assay was performed using the in situ Cell Death Detection Kit (DeadEnd™ Fluorometric TUNEL System, Promega, USA) in accordance with the manufacturer’s protocol. TUNEL-positive cells were considered apoptotic. The fluorescence images were obtained using a fluorescence microscope (Axio Vert A1, Zeiss). The percentage of apoptotic cells was calculated from the ratio of TUNEL-positive cells to total cells under the microscope vision. Three randomly chosen fields were analyzed for each group.

### Transwell migration assay

The migration assay for SC-NPCs was detected in a 24-well plate Boyden chamber with 8-μm pore membrane. The pore membrane of the chamber was coated with polylysine (PL). A total of 1 × 10^4^ cells were placed in the upper chamber of each Transwell. The proliferation medium containing 0.5 μg/mL stromal-derived factor-1 (R&D systems) was added to the lower chamber of the Boyden chamber. After incubated for 24 h, the upper surface of the membrane was gently scrubbed by a cotton swab to remove non-migrating cells. The membranes were fixed in 10% formalin and stained with hematoxylin and eosin. The total numbers of migratory cells were quantified by Image-Pro Plus software (Media Cybernetics).

### Behavioral analyses

The functional abilities of the SCI mice were analyzed weekly at 1, 2, and 3 weeks after surgery using the Basso Mouse Scale (BMS) for locomotion. Mice were observed for motor function in a 30 × 18 cm cage [18].

### Animal care and surgical procedures

All animal experimental procedures were approved by the institutional review board of the Institute of Genetics and Developmental Biology, Chinese Academy of Sciences, and performed in accordance with the Chinese Ministry of Public Health (CMPH) guide for the care and use of laboratory animals. Animals were housed in temperature-controlled environment (~ 22 °C) with alternating 12-h cycles of light and dark. They are free to access food and water. Before the operation, the C57BL/6 mice were anesthetized with ketamine (80 mg/kg) and xylazine (10 mg/kg) injected intraperitoneally. Following the removal of the vertebral lamina, the T10 spinal cord was exposed and then cut off completely using operating scissors. Routine bladder emptying and antibiotic treatment were performed in all animals. In the miR-124 mimics group, the miR-124 mimics were administered using multi-point injections around the SCI site at 5 days after surgery.

### Statistical analysis

Data were statistically analyzed and presented as mean ± SD; differences were considered significant when *p* < 0.05. Factorial analysis of variance (ANOVA) and Student’s *t* test were used for statistical analyses, and these were carried out using statistical software (SPSS version 13.0; SPSS Inc., Chicago, IL, USA).

## Results

### miR-124 enhances Neat1 expression in SC-NPCs and an animal model of SCI

**Fig. 1:**
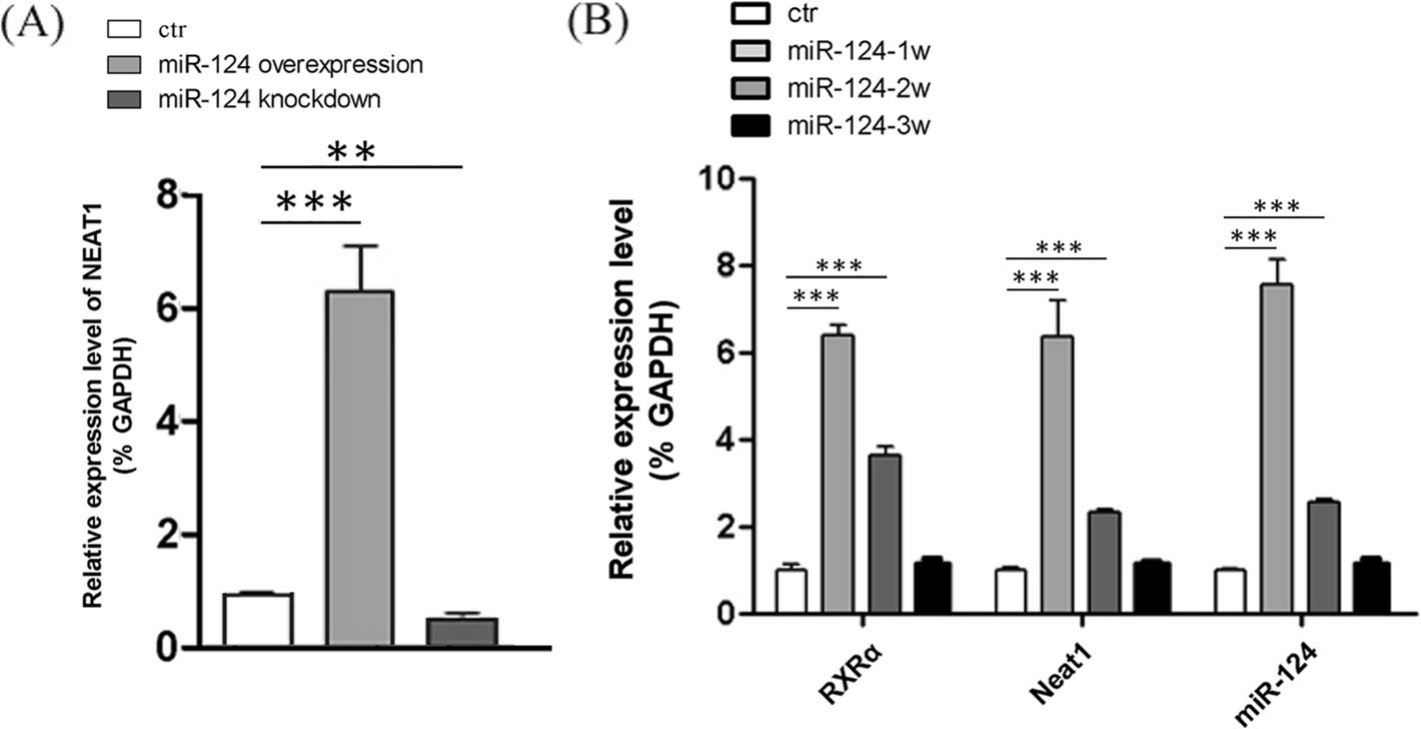
Relative expression of Neat1 gene in the spinal cord neural progenitor cells (SC-NPCs) and spinal cord injury (SCI) animals. Moreover, the relative expression of miR-124 and RXRα were also detected by quantitative real-time PCR (q-PCR). GAPDH expression served as a loading control. **a** The expression of Neat1 in SC-NPCs during differentiation when miR-124 was overexpressed or knocked down. **b** The relative expression of Neat1, miR-124, and RXRα at the injury site after SCI. Expression levels of each gene were normalized to GAPDH. The data are shown as the mean ± SD. *p < 0.05, **p < 0.01, ***p < 0.001

The lncRNA Neat1 had significantly increased expression when miR-124 was overexpressed in SC-NPCs, and significantly decreased expression when miR-124 was knocked down by miR-124 inhibitor (Fig. 1a). Consistent with the cell culture experiments, at 1 or 2 weeks after SCI, miR-124 and Neat1 mRNA expression levels in mice were significantly elevated at the area of injury following the injection of miR-124 mimics compared with controls (Fig. 1b). The expression of miR-124 and Neat1 mRNA was detected using q-PCR. At 2 weeks after the miR-124 injection, the expression of Neat1 was downregulated, accompanied with the decreased expression of miR-124. It has been reported that the nuclear transcription factor RXRα binds to the promoter of Neat1, thus promoting its transcription. The regulatory mechanism was identified by performing ChIP-qPCR and dual-luciferase reporter gene assays [19]. Therefore, we also investigated the expression of RXRα in our SCI animals. As expected, RXRα expression was elevated when miR-124 was overexpressed. Although we did not elucidate a direct regulatory relationship between miR-124, Neat1, and RXRα, the expression trends of Neat1 reflected that its expression was partly regulated by miR-124 in an RXRα-dependent manner.

### miR-124 activates Wnt/β-catenin pathways in SC-NPCs

**Fig. 2:**
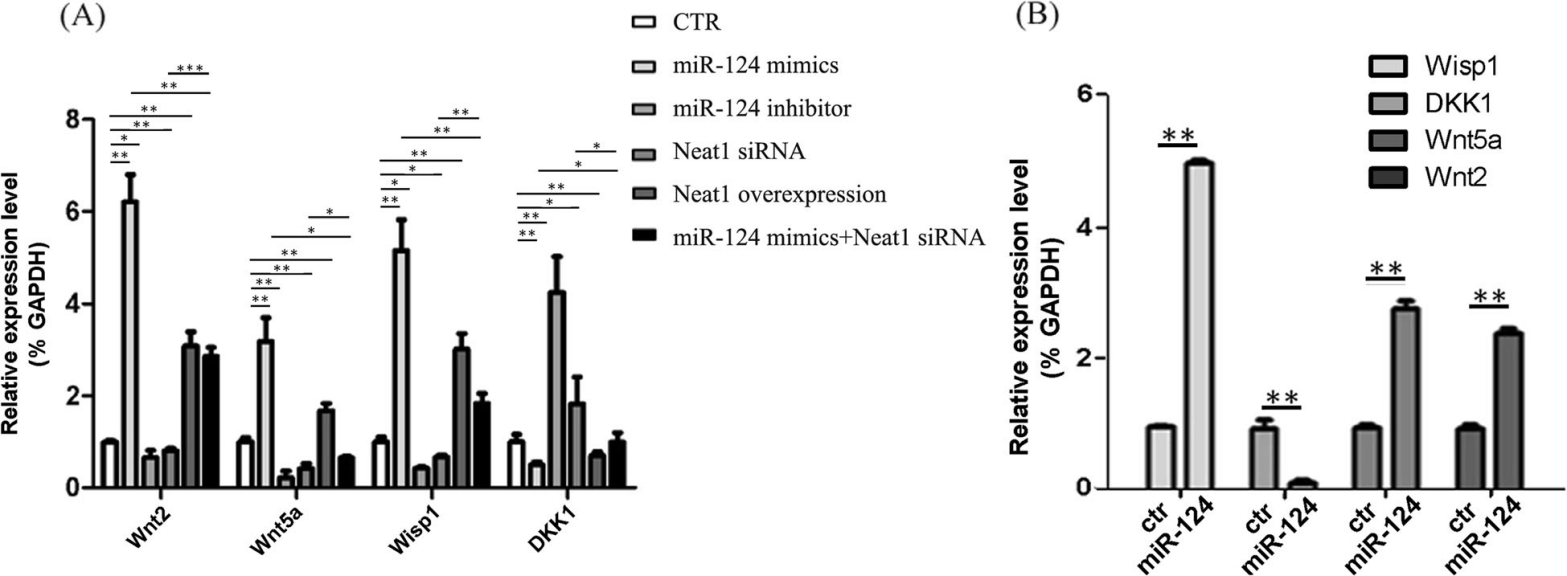
Relative expression of Wnt/β-catenin signaling-related genes (Wisp1, Wnt5a, DKK1, and Wnt2) in each group of SC-NPCs (**a**) and SCI animals (**b**). Expression levels of each gene were normalized to GAPDH. The data are shown as the mean ± SD *p < 0.05, **p < 0.01, ***p < 0.001

The variations of Wnt/β-catenin signaling-related genes induced by miR-124 were measured using q-PCR. Overexpression of mir-124 induced the alteration of many mRNAs involved in regulating the Wnt/β-catenin signaling pathway. Wnt/β-catenin signaling plays key roles in modulating a broad range of cellular activities in stem cells, such as self-renewal, differentiation, apoptosis, and migration [20–23]. To explore the mechanism concerned in the regulation of miR-124 on NPC differentiation, we examined the relative expression of Wisp1, Wnt5a, Wnt2, and DKK1 genes in Wnt/β-catenin signaling pathways. In this study, real-time PCR results indicated that miR-124 elevated the expression of the Wisp1, Wnt5a, and Wnt2, whereas the negative regulator of Wnt/β-catenin signaling gene Dkk1 was downregulated in both SC-NPCs (Fig. 2a) and SCI animals (Fig. 2b). In addition, the miR-124-induced alteration of these four Wnt/β-catenin-related genes showed the same varying tendency when Neat1 was overexpressed or knocked down, indicating that Neat1 was also implicated in activating the Wnt/β-catenin pathways. The combination use of miR-124 mimics and Neat1 siRNA results indicated that the activation of the Wnt/β-catenin pathways was weakened. Taken together, we proposed that the activation of Wnt/β-catenin signaling induced by miR-124 was mediated by the upregulation of Neat1 during SC-NPC differentiation.

### miR-124 and Neat1 promote neuronal differentiation of SC-NPCs both in vivo and in vitro

**Fig. 3:**
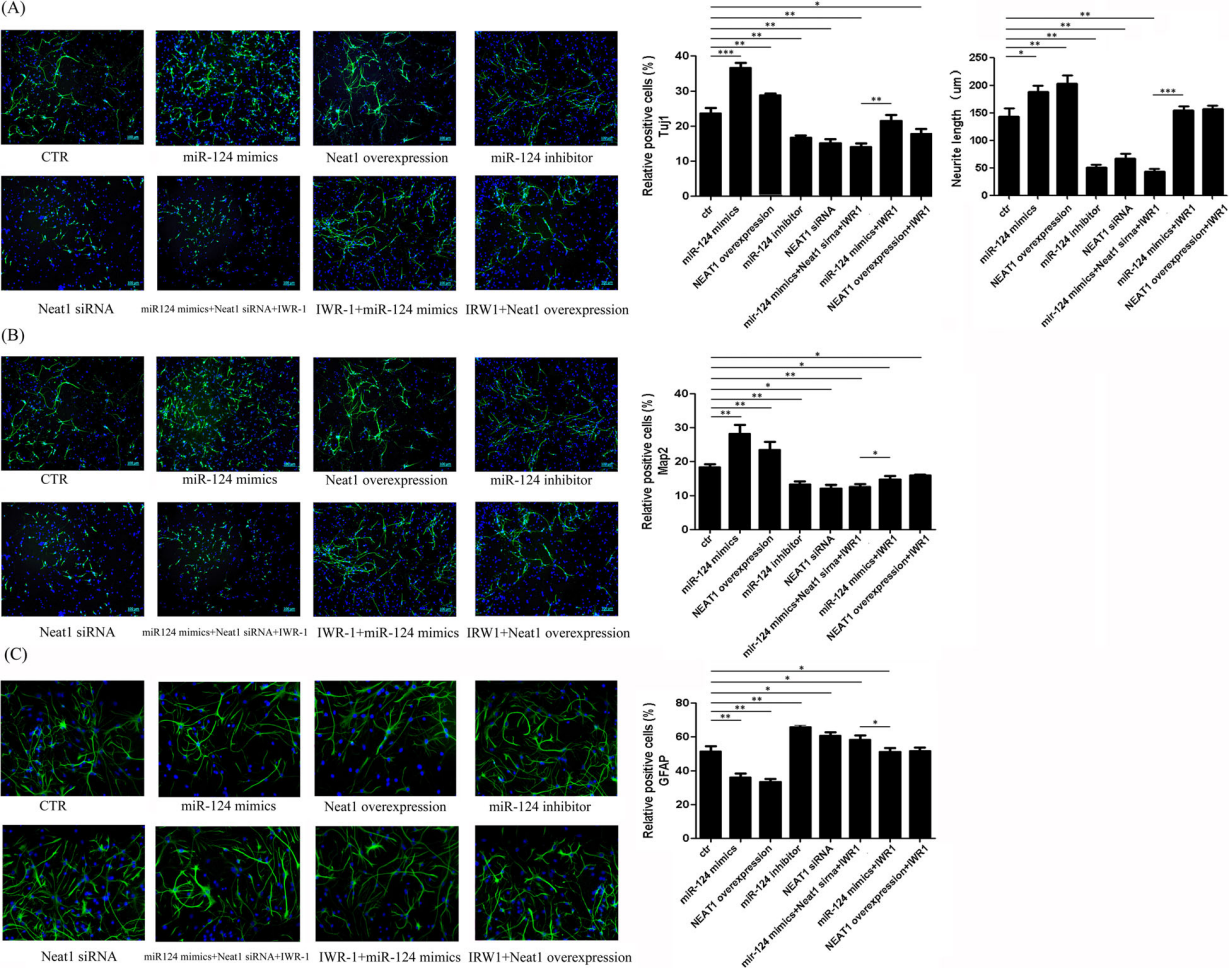
Mir-124/Neat1 promotes neuronal differentiation in cultured SC-NPCs. Representative pictures of immunofluorescence staining of Tuj1^+^ (**a**), Map2^+^ (**b**), and GFAP^+^ (**c**) from each group in cultured SC-NPCs. The graph shows the relative percentages of Tuj1^+^ (**a**, left graph), Map2^+^ (**b**), and GFAP^+^ (**c**) cells in SC-NPCs when cultured in the differentiation medium for 4 days. In addition, we measured the length of neurite extensions from Tuj1-positive cells and then quantified by using Image-Pro Plus software (**a**, right graph). The data are shown as the mean ± SD. *p < 0.05, **p < 0.01, ***p < 0.001

**Fig. 4:**
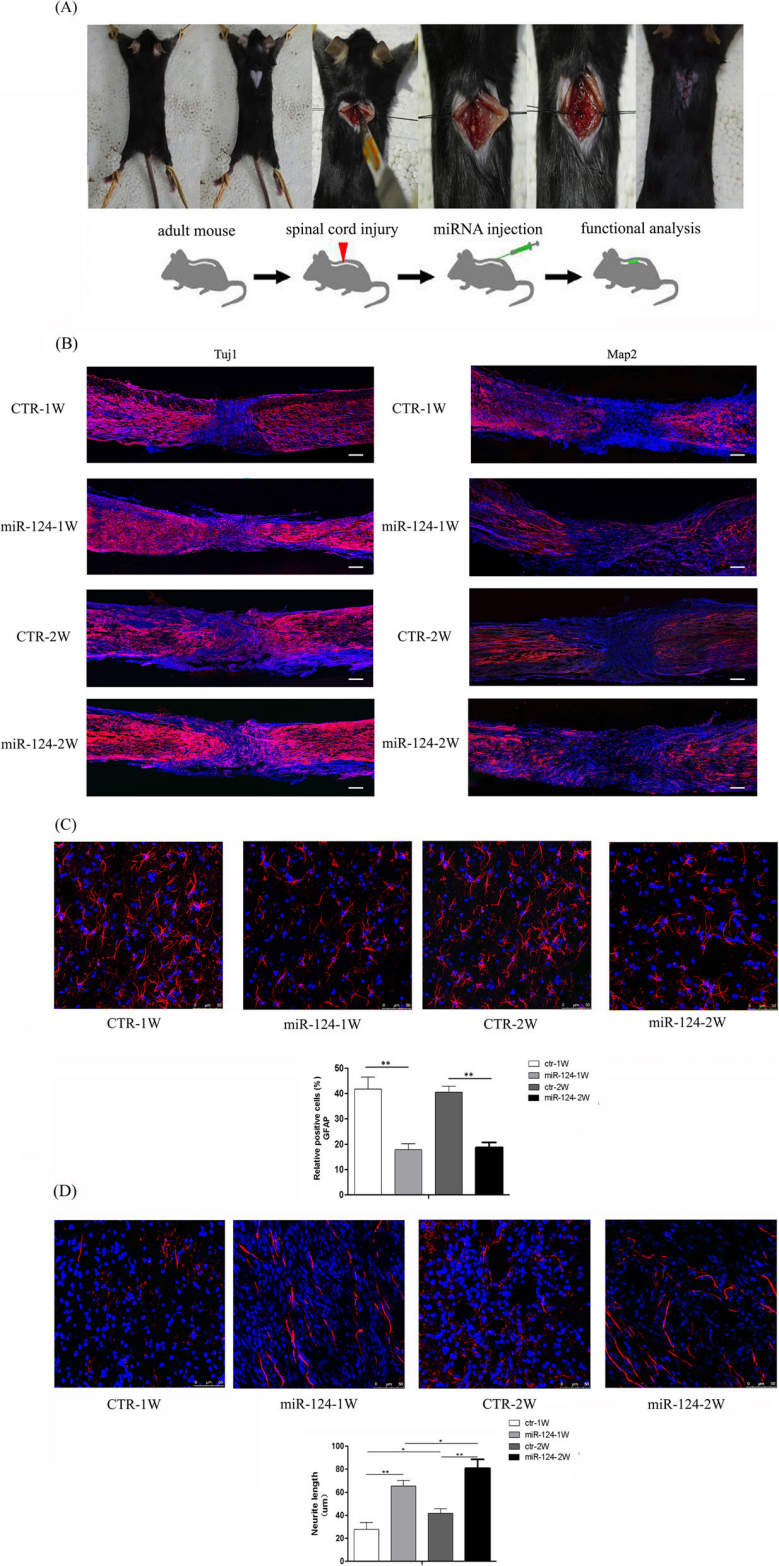
The effect of miR-124 on SCI animals. **a** The surgical procedure used to create the SCI mouse model. Mir-124 mimics were given by multiple injections around the injury site. **b** Overview immunofluorescence staining images of Tuj1^+^ (Left) and Map2^+^ (Right) at the injury site in SCI animal models at 1 and 2 weeks post-injury were shown. **c** The enlarged immunostaining images of GFAP^+^ at the glial scar located in the two sides of broken ends of all groups in SCI animals. The graph shows the relative percentages of GFAP^+^ cells quantified by using Image-Pro Plus software. **d** The enlarged immunostaining images of Tuj1^+^ at the injury site of all groups in SCI animals. The graph shows the neurite length calculated by using Image-Pro Plus software. The data are shown as the mean ± SD. *p < 0.05, **p < 0.01, ***p < 0.001

Immunofluorescence analysis revealed that the percentages of Tuj1^+^ and Map2^+^ cells were significantly elevated when miR-124 was overexpressed, both in cultured SC-NPCs (Fig. 3a, b) and in SCI mice which miR-124 was multiple injected at the site of the injury (Fig. 4a, b). Given that Neat1 and Wnt/β-catenin signaling can be activated by miR-124, we also evaluated their roles in regulating SC-NPC differentiation in vitro. It has been reported that IWR-1 is implicated in the modulation of the Wnt/β-catenin pathways as an inhibitor [24–26]. Cell experiments were randomly divided into eight groups: the control group, miR-124 mimic group, miR-124 inhibitor group, Neat1 siRNA group, Neat1 overexpression group, miR-124 mimic + Neat1 siRNA + IWR-1 group, IWR-1 + miR-124 mimic group, and IWR-1 + Neat1 overexpression group. The results indicated that both miR-124 and Neat1 promote Tuj1 and Map2 expression in cultured SC-NPCs; on the contrary, the expression of GFAP was downregulated in the miR-124/Neat1 overexpression group (Fig. 3c). In accordance with the in vitro results, the percentage of GFAP-positive cells located in glial scar was decreased in miR-124-treated SCI animals (Fig. 4c, Additional file 1). Furthermore, the length of the neurite extension was measured for Tuj1^+^ cells both in cultured SC-NPCs (Fig. 3a) and the injury site of SCI animals (Fig. 4d). The quantified results indicated that miR-124 promoted the neurite length both in vitro and in vivo. And also, Neat1 exerts the same positive function on neurite length as miR-124 in cultured SC-NPCs. The results from the combined application of IWR-1 and miR-124/Neat1 suggest that miR-124/Neat1 may partly rescue the inhibitory effect that IWR-1 has on SC-NPC differentiation. In addition, the rescue by miR-124 of the inhibition caused by IWR-1 was weakened by siNeat1. These results support the hypothesis that Neat1 acts as a mediator between miR-124 and Wnt/β-catenin signaling during SC-NPC differentiation.

### miR-124 and Neat1 inhibit apoptosis in cultured SC-NPCs

**Fig. 5:**
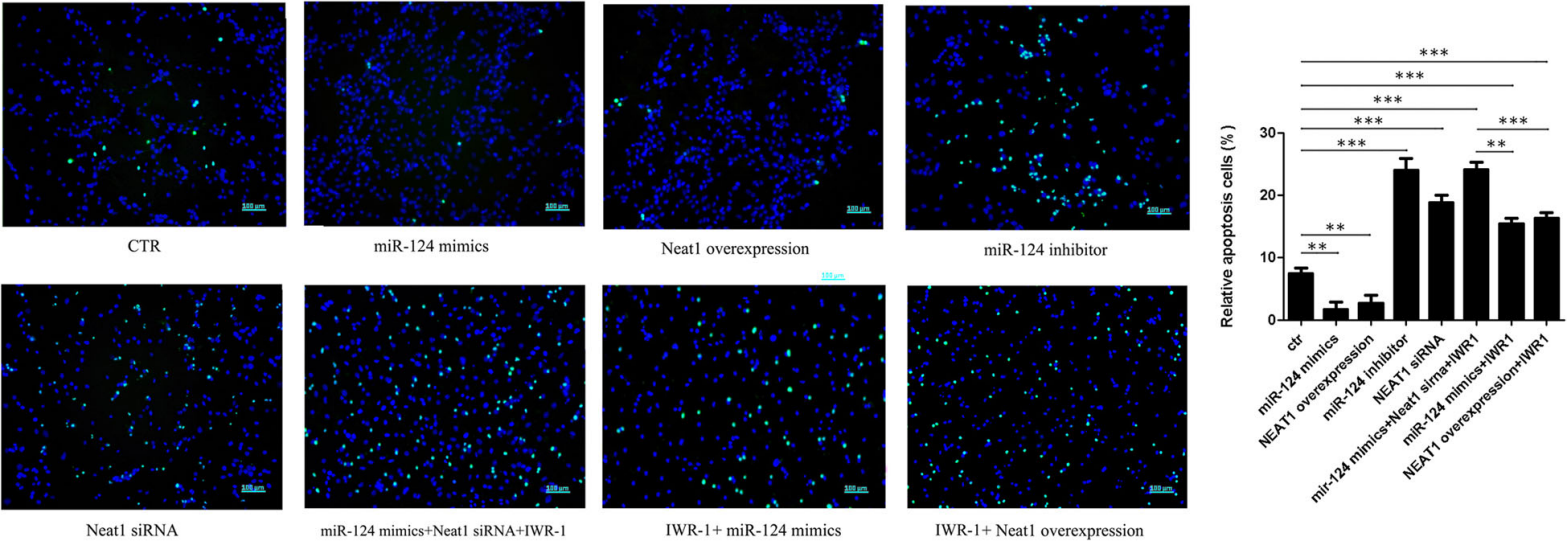
Mir-124/Neat1 inhibits apoptosis in cultured SC-NPCs. Quantitative analysis showing the percentage of apoptotic SC-NPCs in each group when cultured in the differentiation medium for 4 days. The data are shown as the mean ± SD. *p < 0.05, **p < 0.01, ***p < 0.001

TUNEL staining was performed in all eight groups at 96 h after transfection to detect apoptosis. TUNEL-stained photomicrographs of SC-NPCs in all groups are shown in Fig. 5. The results indicated that miR-124 mimics and Neat1 overexpression significantly reduced apoptosis compared with the control group by calculating the percentage of positive apoptotic cells. In contrast, the application of miR-124 inhibitor and siNeat1 led to markedly higher cell apoptosis rates compared with controls. We further investigated the protective effect of miR-124 mimics and Neat1 overexpression on SC-NPC apoptosis when Wnt/β-catenin signaling was inhibited by IWR-1. The TUNEL-positive apoptotic SC-NPCs reduced by IWR-1 treatment were not elevated by miR-124 overexpression when Neat1 was knocked down. Therefore, these data suggest that Neat1 is involved in miR-124-Wnt signaling activation-mediated apoptosis in SC-NPCs and that miR-124 and Neat1 possess a neuroprotective effect by reducing apoptosis in SC-NPCs.

### miR-124 and Neat1 promote migration of cultured SC-NPCs

**Fig. 6:**
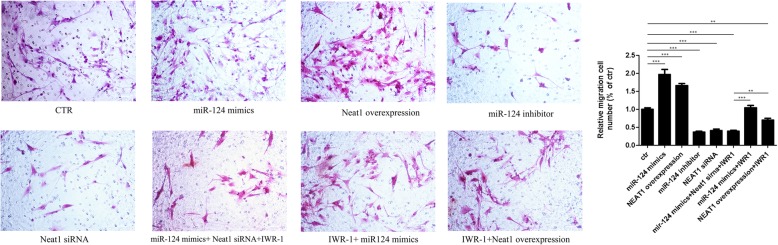
Mir-124/Neat1 facilitates migration in cultured SC-NPCs. Quantitative analysis showing the percentage of migratory SC-NPCs in each group when cultured in the differentiation medium for 4 days. The numbers of migratory cells were normalized to the number of migratory cells in the control group. The data are shown as the mean ± SD. *p < 0.05, **p < 0.01, ***p < 0.001

To detect the effect of miR-124 and Neat1 on SC-NPC migration, we used a Transwell assay to evaluate the migratory ability of SC-NPCs in each of the eight groups. Overexpression of miR-124/Neat1 significantly enhanced the migratory ability of SC-NPCs compared with the control group (Fig. 6). Additionally, the inhibitory effect of IWR-1 on migration was abolished by miR-124/Neat1 overexpression, suggesting that miR-124/Neat1 might regulate the Wnt/β-catenin signaling pathway in SC-NPCs, thus affecting migration. The trends in the variation of migration ability were similar to those of differentiation for all groups.

### miR-124 improves locomotor function and facilitates neural regeneration following SCI

**Fig. 7:**
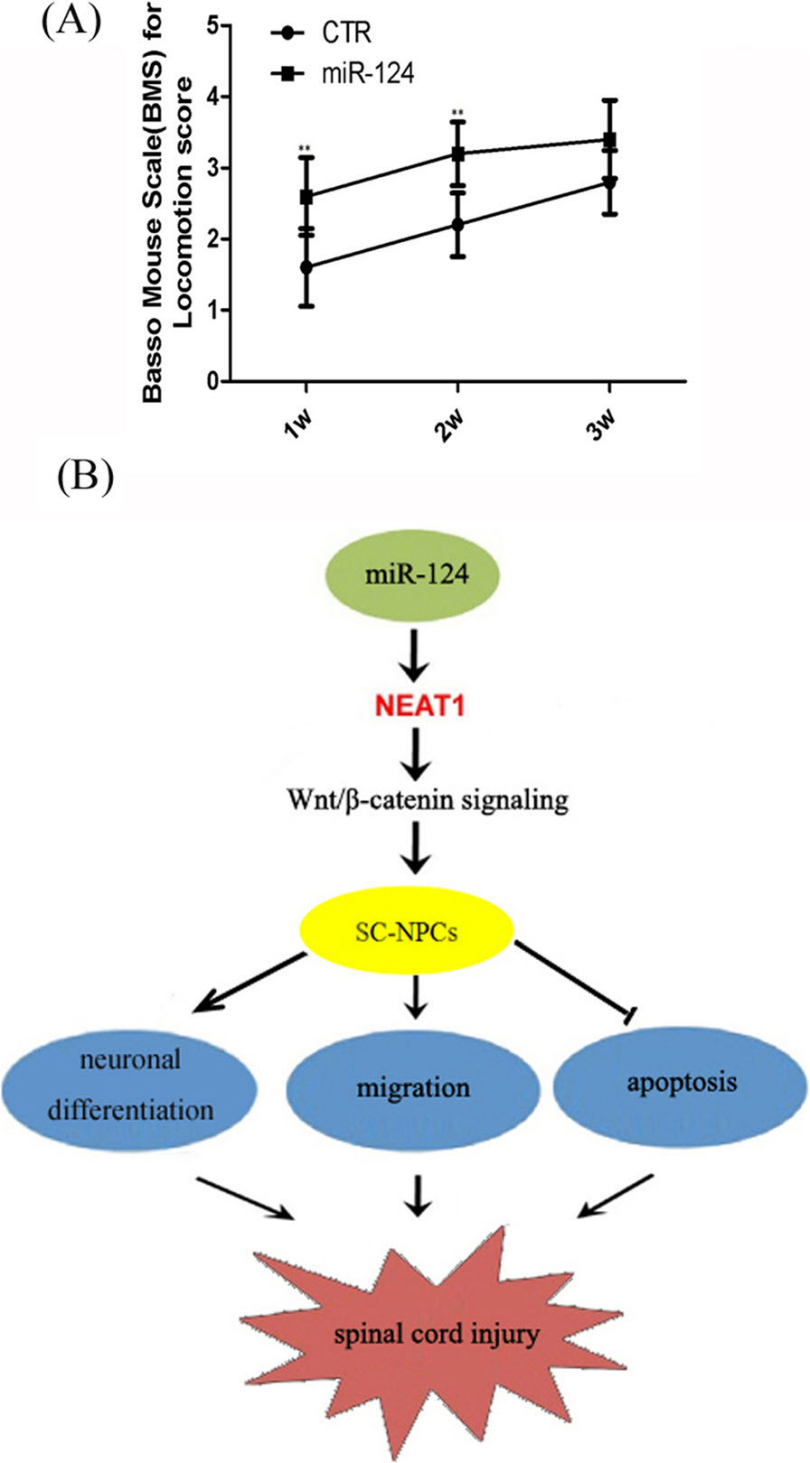
The motor function evaluation of SCI animals and the regulatory mechanism of miR-124 on SC-NPCs. **a** BMS scores were determined at the particular point in time (1, 2, and 3 weeks) after miR-124 treatment. Mir-124-treated animals had significantly higher locomotor scale scores than control mice at 1 and 2 weeks. Error bars represent the mean ± SD of (at least) triplicate experiments. **p < 0.01. **b** Proposed regulatory mechanism of miR-124-NEAT1-Wnt/β-catenin signaling in SC-NPCs. The diagram shows that Neat1 acts as a key mediator between miR-124 and Wnt/β-catenin signaling in regulating SC-NPC behavior

As a sensitive, valid, and reliable measure of locomotion, the Basso Mouse Scale (BMS) was used to evaluate locomotor function recovery in SCI mice. The BMS was performed weekly after injury. All mice were completely paralyzed (BMS score = 0) on the first postoperative day. BMS scores indicated significant differences in locomotor outcomes between the two groups; mice in the miR-124-treated groups achieved higher scores than control animals at 1 or 2 weeks after injection of miR-124. Nevertheless, there were limitations to this study; no continuous improvement was detected at 3 weeks (Fig. 7a). We speculated that this is because a single injection of miR-124 is not enough to promote long-term functional recovery, and multiple dosing should therefore be considered in future studies. Collectively, however, BMS results indicated that miR-124 improves motor abilities and promotes functional recovery in SCI animals.

## Discussion

Recently, many researches paid more attention on SC-NPCs because they were able to promote repair of SCI, which provide a new desire for the treatment of SCI. Many animal researches had demonstrated that SC-NPCs are activated and recruited to the injury site, but these cells tend to differentiate into glial cells rather than neurons [27]. The loss of neurons at the injury site is a major factor influencing functional recovery. Therefore, a major hurdle for future SCI therapies is to understand how to efficiently use the activated endogenous SC-NPCs to treat SCI. Here, we demonstrated that overexpression of miR-124 promoted neuronal differentiation of SC-NPCs both in vitro and in vivo, which brings hope for improving the limited recovery that currently occurs following SCI. As a brain-enriched miRNA, miR-124 has been extensively studied in central nervous system diseases including traumatic brain injury, Parkinson’s disease, cerebral ischemic stroke, and Alzheimer’s disease [1, 28–30]. However, the role of miR-124 in SCI, and the underlying molecular mechanisms of miR-124 in regulating SC-NPCs, is still far from being understood. In our study, we explored the effects of miR-124 on SC-NPC differentiation and as a treatment for the SCI mouse model.

The data from the present study revealed the potential therapeutic effect of miR-124 in SCI mice. All of the mice that received miR-124 injections achieved better functional recovery compared with the control groups at 1 or 2 weeks after injury. The percentage of Tuj1^+^ and Map2^+^ cells was markedly elevated when miR-124 was overexpressed; meanwhile, the percentage of GFAP was reduced significantly both in vitro and in vivo. Considering the importance of apoptosis and migration during spinal regeneration and repair following SCI, TUNEL staining and Transwell assays were also performed on cultured SC-NPCs. The results from these experiments showed that miR-124 promoted migration and suppressed apoptosis. It is generally known that Wnt/β-catenin plays an important role in regulating NSC behavior. The effect of a combined application of miR-124 and IWR-1 further verified that miR-124-mediated SC-NPC differentiation, apoptosis, and migration partly depend on the activation of the Wnt/β-catenin signaling pathways. The precise mechanisms by which miR-124 regulates Wnt/β-catenin signaling need to be confirmed further.

Here, we revealed that the lncRNA Neat1 was elevated when miR-124 was overexpressed both in SCI mice and in cultured SC-NPCs. To date, the functional role of Neat1 in regulating the neuronal differentiation is barely known, so we explored the possible role of Neat1 in the regulation of SC-NPC behavior. Overexpression of Neat1 promoted neuronal differentiation and migration and inhibited apoptosis. In contrast, knockdown studies indicated that loss of Neat1 suppressed neuronal differentiation and migration, but promoted apoptosis. Neat1 is a crucial regulator of Wnt/β-catenin [31]. We therefore hypothesized that Neat1 might boost neuronal differentiation and migration and inhibit apoptosis in a Wnt/β-catenin signaling-dependent manner. Our results indicated that the Neat1-induced promotion of differentiation and migration in SC-NPCs was weakened when combined with IWR-1 treatment. Furthermore, when Neat1 was knocked down, the rescue effect by miR-124 of IWR-1 treatment was diminished. Above all, our results suggest that Neat1 may exert a biological role in mediating the activation of Wnt/β-catenin signaling when miR-124 is overexpressed (Fig. 7b).

Our study has some limitations, however. The potential molecular mechanism that upregulates the expression of Neat1 by miR-124 is still unclear, and further experiments are needed to elucidate the exact regulatory mechanisms of Neat1 by miR-124 in mediating the regulation of Wnt/β-catenin signaling. Moreover, the expression of Neat1 was elevated in both SC-NPCs and SCI animals when miR-124 was overexpressed. The upregulation of the Neat1, which promoted neuronal differentiation and migration and inhibited apoptosis, contributed to the improved functional recovery in mice after SCI. The therapeutic treatment of SCI is always difficult for neurosurgeons in clinic. A better understanding of the miR-124-Neat1-Wnt/β-catenin signaling regulatory pathway will aid in the discovery of new therapeutic targets for patients with SCI.

## Conclusions

Taken together, our results confirm the therapeutic effectiveness of miR-124 on the functional recovery of SCI, supporting the use and feasibility of miR-124 as a future clinical SCI therapy. In addition, our results further point to the role of the lncRNA Neat1 in facilitating neuronal differentiation and migration and inhibiting apoptosis in SC-NPCs. Our study elaborated a novel miR-124-Neat1-Wnt/β-catenin signaling regulatory mechanism for regulating SC-NPC behavior, which may provide a new treatment option for SCI patients.

## Supplementary information

**Additional file 1: Figure S1.**Overview image of immunofluorescence staining (GFAP+) in SCI animal models at 1 and 2 weeks post-injury were shown.

## Data Availability

The datasets supporting the conclusions of this article are included within the article.

## Abbreviations

NSCs: Neural stem cells
LncRNA: Long non-coding RNA
Neat1: nuclear-enriched abundant transcript 1
SC-NPCs: Spinal cord neural progenitor cells
SCI: Spinal cord injury
TUNEL: TdT-mediated dUTP nick-end labeling
EGF: Epidermal growth factor
bFGF: Recombinant human fibroblast growth factor
siRNAs: Small interfering RNAs
PL: Polylysine
BMS: Basso Mouse Scale
q-PCR: Quantitative real-time PCR
ANOVA: Analysis of variance
