# Supplementary material for: LncRNA Neat1 mediates miR-124-induced activation of Wnt/β-catenin signaling in spinal cord neural progenitor cells
Source: Stem Cell Res Ther. 2019 Dec 18;10:400. doi: 10.1186/s13287-019-1487-3 (PMC6921476; doi:10.1186/s13287-019-1487-3)

**Supplement fig 1.**

Overview image of immunofluorescence staining (GFAP+) in SCI animal models at 1 and 2 weeks post-injury were shown.


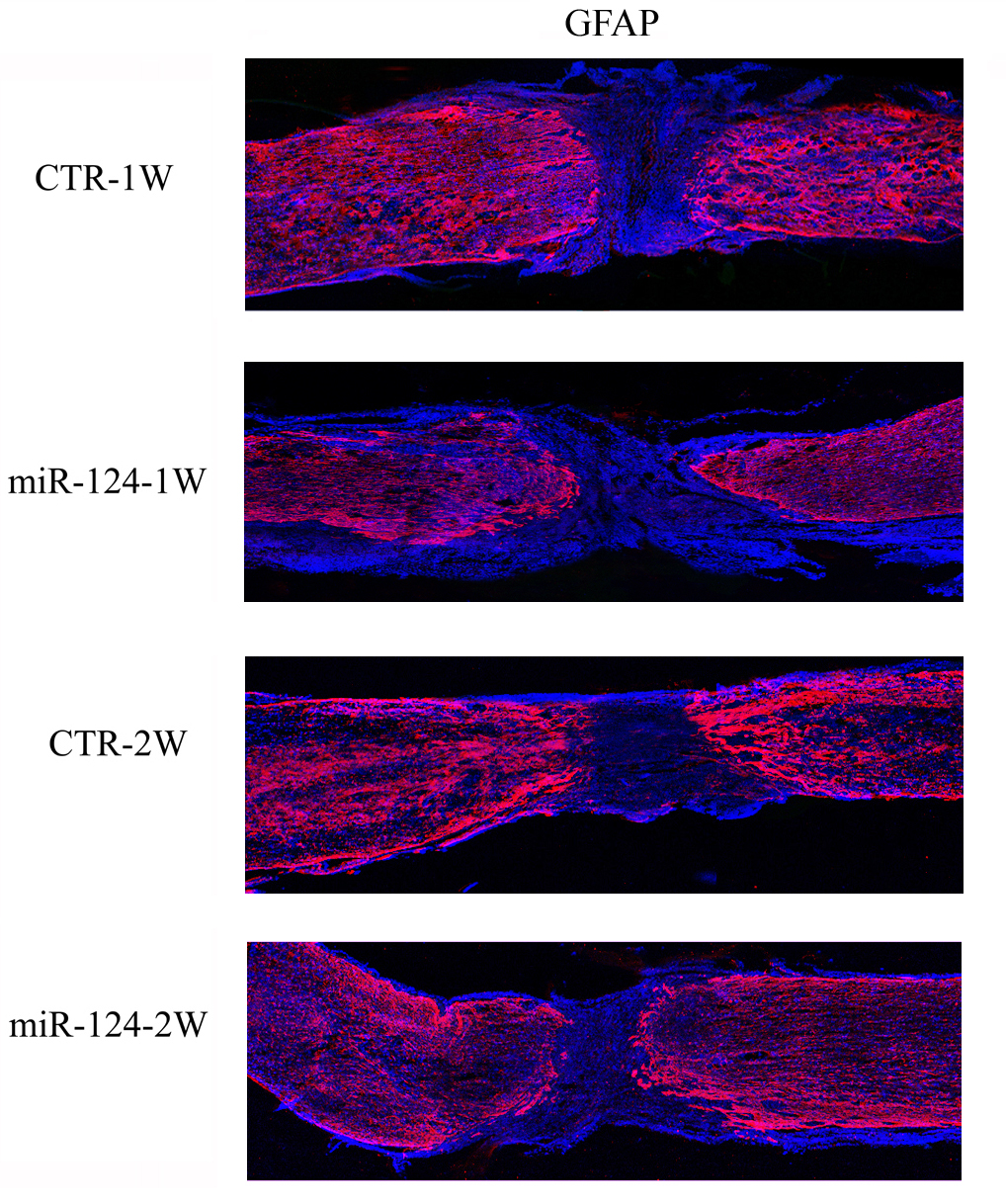

Supplement: Supplementary file 1 — Additional file 1: Figure S1.Overview image of immunofluorescence staining (GFAP+) in SCI animal models at 1 and 2 weeks post-injury were shown. [file 13287_2019_1487_MOESM1_ESM.doc]
